# Close Call

**DOI:** 10.3201/eid1010.AD1010

**Published:** 2004-10

**Authors:** Phebe Hanson

**Keywords:** Keywords: Spanish Flu, Phoebe Hanson, Close Call, Poem, Poetry

All my life my father refused to talk about
his boyhood in Norway. "No," he'd say when
I cajoled him for details. "I'm an American now."
The only thing he'd ever talk about was how he'd
ended up in Minneapolis at Augsburg Seminary,
the story of his "close call," as he referred to it.
He was the only one of his three brothers and sister
who emigrated. "He broke our mother's heart," my aunt
told me when I visited her in Norway many years later.
She gave me the picture she'd taken the day he left, the
day after Christmas, 1920. He's impossibly young, already
wearing his life-long uniform — black suit, vest, white shirt, tie,
ready to go off to America, even if his mother's
heart is breaking, because he had to fulfill a promise
he made when he got the Spanish Flu, summer of 1918.
"Twenty two million people died," he was fond of telling me,
"twice as many as died in World War I, but I didn't die.
When I was choking and close to death, my mother
called the village doctor who performed a tracheotomy
right on our kitchen table and I promised then I'd serve
God forever if He wouldn't let me die. It was a close call."
Close call, I say, echoing my father, now dead these 20 years.
How close he came to being one of the 22 million, how he
almost didn't make it to America, almost didn't spend a
summer in Duluth, preaching at the Norwegian Seaman's
Mission, almost didn't meet my mother whose youth group
was serving coffee and cake after the service, almost didn't
marry her, almost didn't make love with her that warm June
evening of 1927, the night I was conceived, in the white frame
parsonage in Bagley, Minnesota. Close call. Close call.

Phebe Hanson (b. 1928)

From Why Still Dance, Nodin Press, 2003.

Reprinted with author’s permission.

